# Inactivation of Ebola, Nipah, and Lassa viruses in tissue using neutral buffered formalin, MagMAX lysis/binding solution, or TriPure isolation reagent

**DOI:** 10.1038/s41598-025-33750-9

**Published:** 2025-12-31

**Authors:** Katherine A. Davies, Stephen R. Welch, Brian H. Harcourt, Christina F. Spiropoulou, Jessica R. Spengler

**Affiliations:** 1 Viral Special Pathogens Branch, Division of High-Consequence Pathogens and Pathology, Centers for Disease Control and Prevention, Atlanta, GA USA; 2 Zoonotic and Emerging Disease Research Unit, United States Department of Agriculture, National Bio and Agro-Defense Facility, Agricultural Research Service, Manhattan, KS USA

**Keywords:** Virus inactivation, Ebola virus, Nipah virus, Lassa virus, Formalin, Phenol, Guanidinium, Biological techniques, Biotechnology, Microbiology

## Abstract

Samples known or suspected to be infected with high-consequence viruses such as Ebola, Nipah, and Lassa must be handled under high biocontainment. Studies involving animal infections with these pathogens can generate tissues that require downstream analyses, including molecular assays and histopathology, which are more readily performed, or in some cases only feasible, at lower containment levels. Before removal from high containment for analyses at lower containment levels, specimens must undergo validated inactivation procedures. Here, we quantified viral load reduction in tissues infected with these pathogens following treatment with neutral-buffered formalin for 10 min, 1 h, 3 days, or 7 days, and MagMAX lysis/binding solution concentrate or TriPure isolation reagent for 1 or 10 min. To ensure accurate detection of any residual infectious virus, samples were purified through resins or centrifugal filters to reduce reagent cytotoxicity and maximize volume of testable material. We demonstrated effective inactivation (≥ 4 log_10_ reduction) of all three pathogens and quantified log-reduction values over multiple timepoints. These findings provide validation data to support safe handling of infectious tissues for research, field studies, and outbreak response.

**Supplementary Information:**

The online version contains supplementary material available at 10.1038/s41598-025-33750-9.

## Introduction

Effective inactivation procedures are essential when working with high-consequence viruses to protect both personnel and the environment, enable downstream analyses in lower-containment level laboratories, and ensure compliance with regulatory requirements. Thorough evaluation of inactivation methods for infected tissues is particularly important for diagnostic applications, animal model development, and preclinical studies of medical countermeasures. Inactivation methods include physical inactivation (e.g., ionizing radiation)^1,2^, thermal inactivation (e.g., heat)^3,4^, or chemical inactivation (e.g., chaotropic, oxidative, and fixative reagents)^3^. The choice of inactivation procedure is based on proven inactivation efficacy for the specific agent and matrix, and compatibility with intended downstream assays (analyte preservation and inhibitor management).

Effective inactivation methods are generally expected to demonstrate ≥ 4 log_10_ reduction in viral titers (equivalent to ≥ 99.99% reduction) and no recovery of infectious virus^5^. To meet these requirements, inactivation studies should consider (i) methods to minimize virucidal-reagent cytotoxicity, and (ii) selection of appropriate tissue specimens containing high viral titers and with sufficient material available for analysis. Virucidal reagents are often cytotoxic in cell culture, complicating accurate detection of residual infectious virus^6^. Reducing cytotoxicity is therefore critical and can be achieved using methods such as dilution, dialysis, filtration, or purification resins^6–8^. In addition, starting titers should be at least 6 log_10_ per gram of tissue, requiring identification of high-titer infectious tissue. Adequate sample volume is also required; however, in small animal models (e.g., mice), organ size may present limitations.

The U.S. Federal Select Agent Program requires in-house studies to be performed to verify inactivation conditions^9^. The virucidal efficacy of inactivants has been assessed for risk group-4 pathogens, including Ebola (EBOV), Marburg (MARV), Nipah (NiV), Lassa (LASV), and variola (VARV) virus. For EBOV, MARV, and LASV, chemical inactivation using guanidinium isothiocyanate was found to be effective when combined with ethanol^7,10,11^. For EBOV, MARV, NiV, and VARV, phenol- and aldehyde-based virucidal compounds also have demonstrated efficacy^7,12–15^. To date, most inactivation validation of high-consequence pathogens with aldehyde- or guanine thiocyanate-based inactivants focus on infectious cell culture supernatants, with few studies evaluating serum^16^, plasma^17^, blood^18^, cell monolayers and pellets^8,16,17,19^. Inactivation of high-consequence viral pathogens in these sample types has also been evaluated using other chemicals and disinfectants^3^. However, inactivation efficacy data on animal tissue samples remain limited^7,12,13,17,19^, likely due to additional technical challenges and scarcity of appropriate samples for testing.

Here, we show that purification resins and/or centrifugal filters provide a straightforward and effective way to reduce the cytotoxicity of neutral-buffered formalin (NBF), guanidinium thiocyanate-based MagMAX lysis/binding solution concentrate (MagMAX), and phenol-based TriPure isolation reagent (TriPure), allowing rapid processing, maintaining the fidelity of contact times, and maximizing the volume of sample permissible for viability testing. Using these methods, we demonstrate effective inactivation of virus in tissue and determined log-reduction values over multiple timepoints for three high-consequence pathogens: EBOV, NiV, and LASV. These data provide assurance for the use of these viral inactivation procedures to support laboratory research, field studies, and outbreak response.

## Results

### Determination of virus-specific target tissues from experimentally infected rodents for inactivation studies of Ebola, Nipah, and Lassa viruses

For inactivation validation, tissue samples should be (i) of sufficient titer (> 6.0 log_10_ TCID_50_/g) to achieve at least a 4 log_10_ reduction (99.99%) in virus titer, (ii) of sufficient size to replicate those used in actual laboratory conditions, and (iii) maintain integrity after freeze–thaw. To determine the most appropriate tissue type for inactivation studies, we first quantified infectious virus from archived tissues positive for viral RNA (vRNA) by RT-qPCR (Table 1). These samples were collected from historic studies, including mouse-adapted EBOV-infected mice (variant Mayinga; liver, spleen, gonads, eye, and brain), NiV-infected hamsters (strain Malaysia: liver, kidney, lung, and brain; strain Bangladesh: lung, and brain), and LASV-infected guinea pigs (strain Josiah; liver, spleen, and lung) that met endpoint criteria.

**Table 1:** Characterization of Ebola, Nipah, and Lassa virus infectious titers in tissues from experimentally infected rodents.

		Liver	Spleen	Testes	Sem. Ves	Ovary	Uterus	Kidney	Lung	Eye	Brain
Mouse-adapted Ebola virus (Mice)	n =	40	40	18	16	22	22	0	0	27	34
	Minimum	4.90	4.61	3.56	3.00	3.71	3.81	−	−	2.32	1.89
	Maximum	8.81	8.30	6.95	7.40	7.92	9.24	−	−	5.82	7.43
	Mean	8.20	7.64	6.17	6.55	7.18	8.09	−	−	4.81	6.10
	Median	8.15	7.57	5.62	5.69	6.98	7.44	−	−	4.24	5.43
Nipah virus (Hamsters)	n =	14	0	0	0	0	0	9	31	0	12
	Minimum	2.53	−	−	−	−	−	2.64	2.23	−	2.49
	Maximum	3.83	−	−	−	−	−	5.16	7.50	−	5.21
	Mean	3.21	−	−	−	−	−	4.55	6.50	−	4.56
	Median	2.86	−	−	−	−	−	3.46	5.43	−	3.91
Lassa virus (Guinea pigs)	n =	13	13	0	0	0	0	0	5	0	0
	Minimum	3.96	5.35	−	−	−	−	−	6.73	−	−
	Maximum	7.08	8.30	−	−	−	−	−	8.28	−	−
	Mean	6.41	7.49	−	−	−	−	−	7.84	−	−
	Median	5.29	6.77	−	−	−	−	−	7.79	−	−

Tissue titers (log_10_ TCID_50_/g) of infectious Ebola virus (mouse-adapted, variant Mayinga), Nipah virus (NiV; strain Malaysia or Bangladesh), Lassa virus (strain Josiah) in terminal animals (i.e., those meeting endpoint criteria or succumbing to infection). Data represents quantification of infectious virus in over 300 unique tissue specimens positive for viral RNA by RT-qPCR. NiV specimens comprised tissues from hamsters infected with strain Malaysia [kidney, n = 9; lung, n = 15; brain, n = 10] or strain Bangladesh [n = 9; lung, n = 16; brain, n = 2]. −, indicates analyses not performed. Sem. Ves, seminal vesicle.

High levels (> 6.0 log_10_ TCID_50_/g) of infectious virus were detected in liver (EBOV, LASV), spleen (EBOV, LASV), lung (NiV, LASV), and reproductive organs (EBOV). In general, median titers were lower in NiV-Bangladesh infected hamsters than NiV-Malaysia-infected hamsters in both lung [NiV Malaysia (n = 15): 6.0 log_10_ TCID_50_/g (range: 4.0–7.5 log_10_ TCID_50_/g); NiV Bangladesh (n = 16): 4.5 log_10_ TCID_50_/g (range: 2.2–6.3 log_10_ TCID_50_/g)] and brain [NiV Malaysia (n = 10): 4.4 log_10_ TCID_50_/g (range: 3.4–5.2 log_10_ TCID_50_/g); NiV Bangladesh (n = 2): 2.7 log_10_ TCID_50_/g (range: 2.5–2.8 log_10_ TCID_50_/g)].

For subsequent evaluations, target tissues were selected based on titer (> 6 log_10_ TCID_50_/g), organ size, integrity, and sample availability. Tissue samples, including gonads, kidneys, and eyes, were determined to be of insufficient size for further testing. Furthermore, brain samples did not maintain integrity after one freeze–thaw cycle and therefore could not be considered for evaluation. Liver was identified as a target tissue for EBOV, with 95% tested tissues having titers > 6.0 log_10_ TCID_50_/g. Lung was identified as the primary target tissue for NiV; however, only 32% tested samples had titers > 6.0 log_10_ TCID_50_/g (NiV-Malaysia: n = 9 and NiV-Bangladesh: n = 1). To expand sample availability for NiV, we also included tissues with titers > 5.0 log_10_ TCID_50_/g, which comprised 52% of tested samples (NiV-Malaysia: n = 12 and NiV-Bangladesh: n = 4). Lung and spleen were identified as target tissues for LASV; 67% of lung and 83% of spleen tissues tested had titers > 6.0 log_10_ TCID_50_/g.

### Purification resins and filters effectively reduce cytotoxicity of neutral buffered formalin, MagMAX lysis/binding solution concentrate, and TriPure isolation reagent

**Fig. 1:**
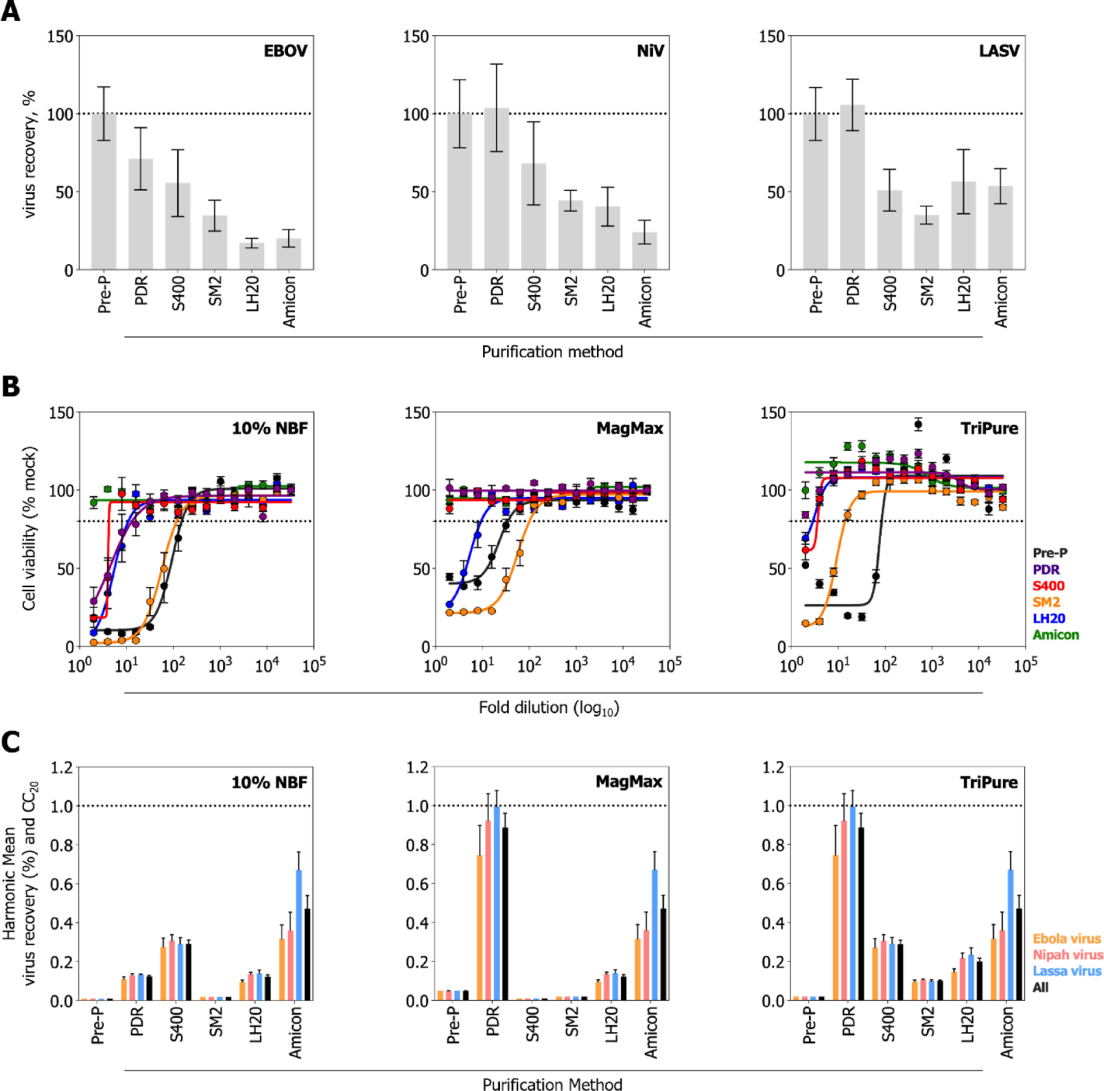
Purification resins and centrifugal filters can effectively reduce cytotoxicity of neutral buffered formalin, MagMAX Lysis/Binding Solution Concentrate, and TriPure isolation reagent while maintaining high viral recovery. (**A**) Virus recovery after purification through resins (Pierce detergent removal [PDR], Sephacryl S400 [S400], Sephadex LH20 [LH20], and Bio-Beads SM-2 Adsorbents [SM2]) and Amicon Ultra-0.5 Centrifugal Filter Units, 50 kDa (Amicon) were evaluated. Viral titers were quantified pre- (Pre-P) and post-purification. Viral recovery is presented as a percentage of the average pre-purification titers. The bar indicates the mean value of six independent replicates, and the error bars indicate the standard error of the mean. The dotted line indicates 100% recovery, compared to the average of the pre-purification titers. (**B**) Purification resins (PDR, S400, LH20, and SM2) and Amicon centrifugal filters [50 kDa] (Amicon) were evaluated for effectiveness at reducing cytotoxicity from liver and lung samples treated 1:10 with 10% neutral buffered formalin (NBF), MagMAX Lysis/Binding Solution Concentrate (MagMAX), and TriPure isolation reagent (TriPure). Concentration–response curves were performed on Vero E6 cells using twofold serial dilutions. Control samples to demonstrate toxicity pre-purification were also included. Cell viability was assessed 72 h post-treatment and normalized to untreated control cells. Each point represents the mean of eight samples, comprising four technical replicates each from treated liver and lung tissue; error bars indicate the standard error of the mean. The dotted line indicates 80% cell viability. (**C**) To determine the most appropriate method to reduce cytotoxicity while maintaining high viral recovery, the harmonic mean $[\frac{2ab}{a+b}]$ was calculated from percentage viral recovery (n = 6) and cytotoxic concentration 20% (CC_20_). The bar indicates the mean, and the error bars indicate the standard error of the mean.

Virucidal reagents are often cytotoxic, complicating detection of residual infectivity post-treatment. To reduce cytotoxicity and support accurate inactivation assessment, we evaluated four purification resins (Pierce detergent removal resin [PDR], Sephacryl S400HR [S400], Sephadex LH20 [LH20], and Bio-Beads SM-2 Adsorbents [SM2]) and a centrifugal filter unit (Amicon Ultra-0.5 Centrifugal Filter Unit, 50 kDa [Amicon]) for cytotoxicity reduction and virus recovery post-purification in tissues collected from rodents experimentally infected with EBOV, NiV, or LASV. PDR resin was the most efficient purification method for virus recovery (Fig. 1A) and was effective at reducing cytotoxicity with MagMAX and TriPure (Fig. 1B). However, PDR resin exhibited poor cytotoxicity reduction with 10% NBF. S400 resin effectively reduced cytotoxicity from MagMAX but not from TriPure or NBF, and was not as efficient as PDR for virus recovery. SM2 and LH20 resins were not effective in reducing cytotoxicity from MagMAX, TriPure, or NBF, and demonstrated poor virus recovery. Amicon centrifugal filter units effectively reduced the cytotoxicity of all reagents evaluated here, but demonstrated poor virus recovery.

To determine the optimal inactivant-specific method for minimizing cytotoxicity while maximizing virus recovery, harmonic means of the 20% cytotoxic concentration (CC_20_) versus percentage viral recovery were calculated (Fig. 1C). For NBF-treated tissues, Amicon filters were determined to be optimal due to their effective reduction of cytotoxicity, as complete removal of NBF is essential; even trace amounts of residual NBF can adversely affect cell cultures. For MagMAX- and TriPure-treated tissue, PDR resin was determined to be optimal based on strong performance in both cytotoxicity reduction and virus recovery.

### Low-titer Ebola, Nipah, and Lassa virus can be detected using cell-culture-based virus titration and blind passage methods

**Fig. 2:**
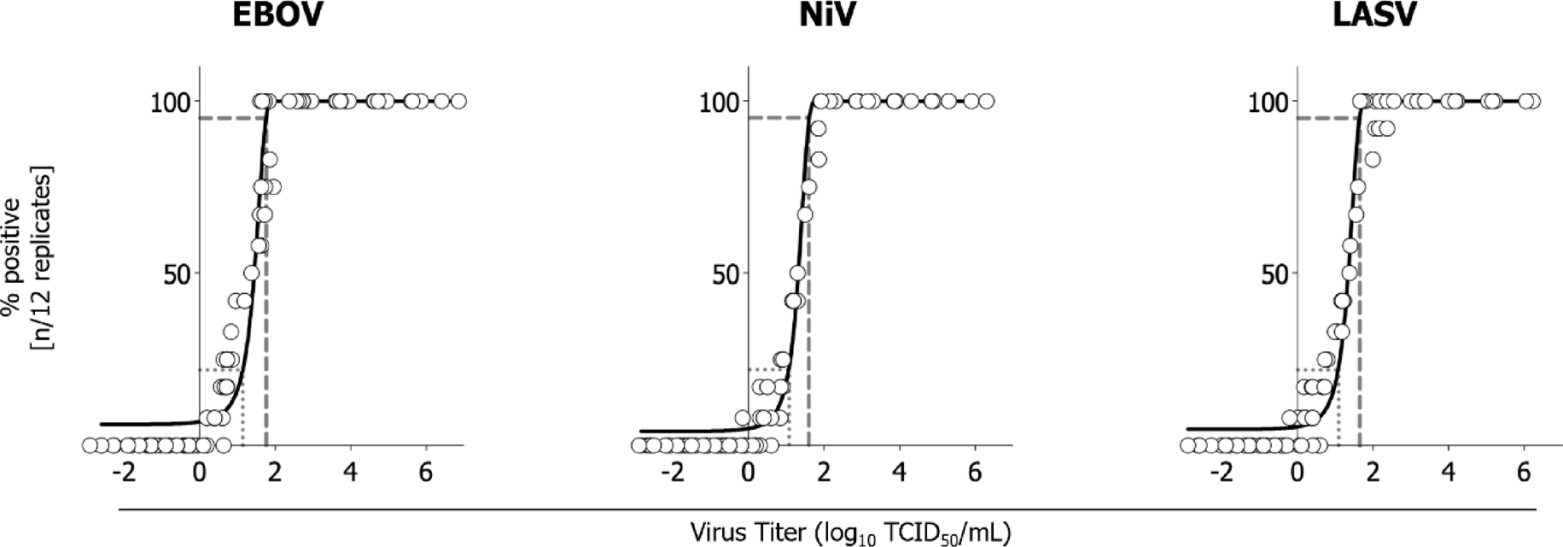
Probit curves to determine the limit of detection and minimal detectable concentration for Ebola, Nipah, and Lassa viruses. Virus titration data for EBOV, NiV, and LASV were analyzed using a probit regression model to determine the limit of detection and the minimal detectable concentration. Virus titer is plotted against the percentage of positive replicates (n/12). The dashed line indicates the limit of detection (LOD; the lowest concentration of infectious virus predicted to be positive in 95% of replicates) and the dotted line indicates the minimum detectable concentration (MDC; the lowest concentration of infectious virus predicted to yield one positive replicate out of 12 replicates).

To confirm that low levels of infectious viruses are detectable by the assays used herein, we assessed the lowest detectable virus titers and established limit of detection (LOD) values for each virus species. Working stocks, serially diluted to approximately 6.0–0.1 log_10_ TCID_50_/mL, were titrated or inoculated blind (without a predetermined titer) onto Vero E6 cells for EBOV or Vero cells for NiV and LASV. Detection of infectious virus by titration (quantification) or in flasks (qualification) was recorded. Low levels of infectious virus (0.4–0.7 log_10_ TCID_50_/mL) were detectable by passage and titration for all viruses (Table 2). Using probit analysis, we determined the LOD, which is defined as the lowest concentration of infectious virus that would be detectable in 95% of replicates. The LOD for EBOV, NiV, and LASV was 1.77, 1.61, and 1.64 log_10_ TCID_50_/mL, respectively (Fig. 2). In addition, we calculated the minimum detectable concentration (MDC) for TCID_50_ assays performed using 12 technical replicates. The MDC is defined as the lowest concentration of infectious virus that could be detected in the TCID_50_ assay, where ≥ 1 replicate out of 12 replicates would be positive. The MDC for EBOV, NiV, and LASV was 1.15, 1.07, and 1.08 log_10_ TCID_50_/mL, respectively.

**Table 2:** Viral titration and blind passage detect low titers of Ebola, Nipah, and Lassa viruses.

Dilutions	Ebola virus			Nipah virus			Lassa virus		
	Actual titer (log_10_ TCID_50_/mL)	Detectable by titration	Detectable by passage	Actual Titer (log_10_ TCID_50_/mL)	Detectable by titration	Detectable by passage	Actual titer (log_10_ TCID_50_/mL)	Detectable by titration	Detectable by passage
Ten-fold	6.76	Yes (2/2)	Yes (2/2)	5.98	Yes (2/2)	Yes (2/2)	6.15	Yes (2/2)	Yes (2/2)
	5.62	Yes (2/2)	Yes (2/2)	4.85	Yes (2/2)	Yes (2/2)	5.11	Yes (2/2)	Yes (2/2)
	4.86	Yes (2/2)	Yes (2/2)	3.86	Yes (2/2)	Yes (2/2)	4.10	Yes (2/2)	Yes (2/2)
	3.73	Yes (2/2)	Yes (2/2)	3.24	Yes (2/2)	Yes (2/2)	3.38	Yes (2/2)	Yes (2/2)
	2.60	Yes (2/2)	Yes (2/2)	1.92	Yes (2/2)	Yes (2/2)	2.32	Yes (2/2)	Yes (2/2)
Two-fold	2.19	Yes (2/2)	Yes (2/2)	1.56	Yes (2/2)	Yes (2/2)	1.70	Yes (2/2)	Yes (2/2)
	1.70	Yes (2/2)	Yes (2/2)	1.48	Yes (2/2)	Yes (2/2)	1.64	Yes (2/2)	Yes (2/2)
	1.64	Yes (2/2)	Yes (2/2)	1.25	Yes (2/2)	Yes (2/2)	1.40	Yes (2/2)	Yes (2/2)
	1.19	Yes (2/2)	Yes (2/2)	1.08	Yes (2/2)	Yes (2/2)	1.31	Yes (2/2)	Yes (2/2)
	0.72	Yes (2/2)	Yes (2/2)	0.40	Yes (2/2)	Yes (2/2)	0.59	Yes (2/2)	Yes (2/2)
	0.40	Yes (2/2)	No (0/2)	≤ 0.10	Yes (1/2)	Yes (2/2)	0.40	Yes (2/2)	Yes (2/2)
	≤ 0.10	Yes (1/2)	No (0/2)	n.d	No (0/2)	Yes (1/2)	≤ 0.10	Yes (1/2)	Yes (1/2)

Ebola, Nipah, and Lassa virus stocks were serially diluted in a range spanning approximately 6.0–0.1 log_10_ TCID_50_/mL. Detection of infectious virus was assessed by either TCID_50_ (12 technical replicates) or blind passage onto flasks of cells. Assays were performed in duplicate. Actual titers were calculated using the Reed-Munch method, or, where low levels of virus were detected, using the Taylor Formula. Titers are reported as ≤ when infectious virus was not detected in at least one sample, or as "n.d." when infectious virus was not detected in any sample. Detection of infectious virus by blind passage was determined by RT-qPCR, with a greater than tenfold increase in vRNA (equivalent to a decrease in C_t_ value >3.3) indicating the presence of infectious virus.

### Ebola, Nipah, and Lassa viruses in tissues are effectively inactivated by neutral-buffered formalin, MagMAX Lysis/binding solution concentrate, and TriPure isolation reagent

**Fig. 3:**
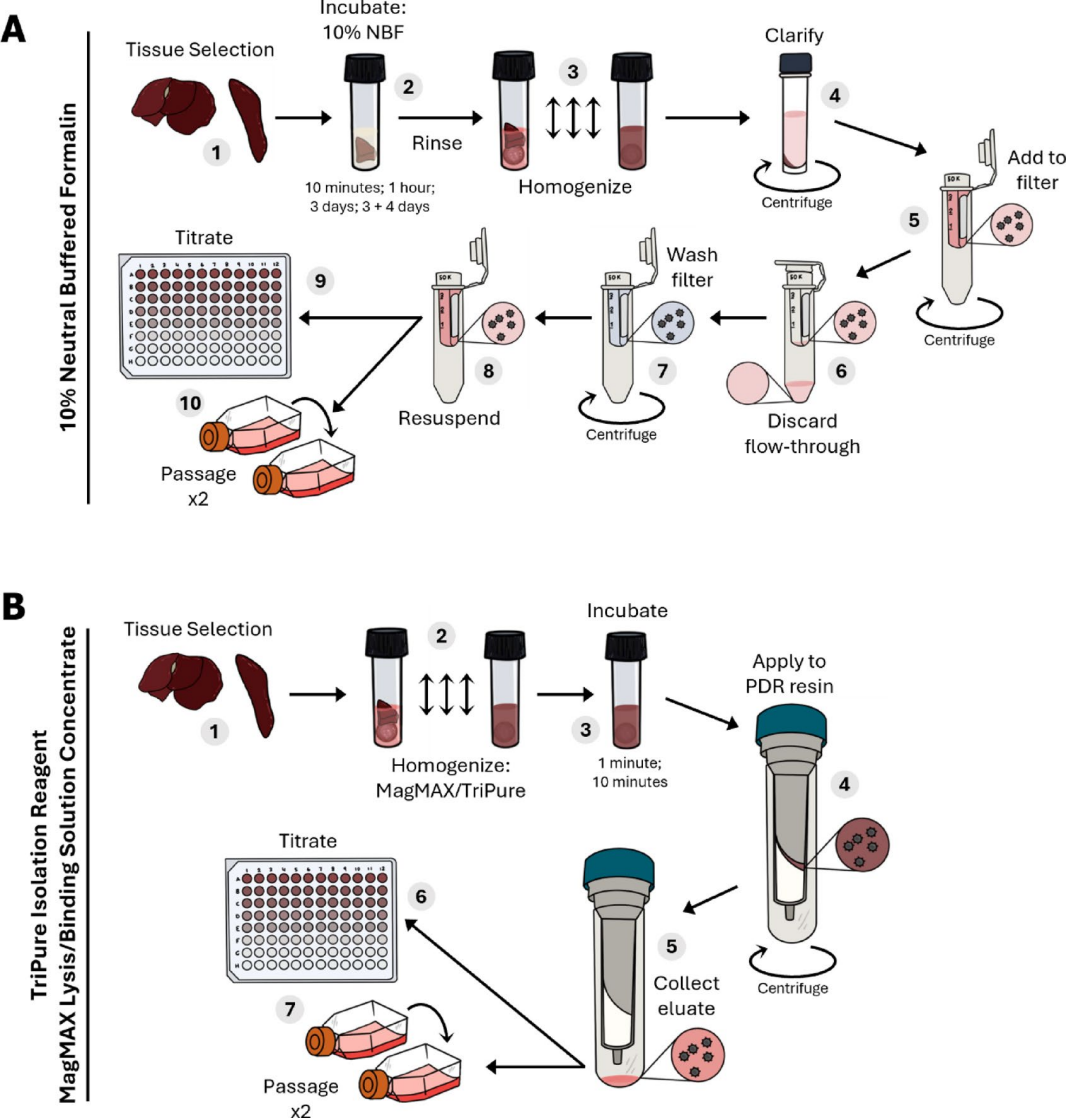
Inactivation evaluation workflow. Workflow demonstrating steps to evaluate inactivation of tissues using either (**A**) 10% neutral buffered formalin (NBF), or (**B**) MagMAX Lysis/Binding solution concentrate (MagMAX) or TriPure Isolation Reagent (TriPure). (**A**) 1, target tissues and specimens selected to maximize evaluation of inactivation efficacy (i.e., sufficient titer and tissue availability), trimmed and weighed; 2, tissues incubated with 10% NBF for 10 min, 1 h, 3 days or 7 days (with complete product replacement on day 3); 3, fixed tissue rinsed in PBS and homogenized in DMEM supplemented with 10% FBS and 2× antimycotic antibiotic; 4, homogenate clarified by centrifugation; 5, clarified homogenate added to Amicon Ultra-0.5 Centrifugal Filter Units [50 kDa]; 6, Amicon columns centrifuged and flow-through discarded; 7, filters washed three times in PBS; 8, filtrate resuspended in DMEM supplemented with 10% FBS and 2× antimycotic antibiotic; 9–10, titration and blind passage of purified material in parallel, supernatants from flasks were passaged again if no virus was detected in the first passage. (**B**) 1, target tissues and specimens selected to maximize evaluation of inactivation efficacy (i.e., sufficient titer and tissue availability), trimmed and weighed; 2 tissues immediately homogenized in MagMAX or TriPure (1:4 or 1:10 [w/v]); 3, homogenate incubated for 1 min, or 10 min; 4, homogenate added to centrifuge columns containing Pierce detergent removal (PDR) resin; 5, columns centrifuged and eluate collected; 6–7, titration and blind-passage of purified material, supernatants from flasks were passaged again if no virus was detected in the first passage. Graphics were created in Procreate (v5).

To assess the efficacy of EBOV, NiV, and LASV inactivation by NBF, MagMAX, or TriPure, tissues with known viral titers from experimentally infected rodents were treated with each inactivant for predetermined contact times and then processed using protocols optimized in this study for virus recovery and cytotoxicity reduction (Amicon filter units for NBF and PDR resin spin columns for MagMAX and TriPure). The processed samples were subsequently titrated to quantify infectious virus and, in parallel, subjected to blind passaging to determine whether any infectious virus remained following inactivation (Fig. 3). For each condition (tissue, inactivant, ratio, and contact time), tissues from three to six individual animals were used.

Tissue samples were treated with a 1:10 (w/v) ratio 10% NBF for 10 min, 1 h, 3 days, or 7 days (with a complete NBF replacement on day 3) (Fig. 3A). No infectious virus was detected after ≥ 1 h of treatment in any sample. After 10 min contact time, no infectious virus was detected in EBOV-infected liver or NiV-infected lung, with > 4 log_10_ reductions observed (Table 3). However, after a 10 min treatment, infectious LASV was detected from both spleen and lung tissues, with 1.9- and 3.1-log_10_ TCID_50_/g average reductions observed, respectively.

**Table 3:** 10% neutral buffered formalin effectively inactivates Ebola, Nipah, and Lassa viruses in tissues.

10% Neutral buffered formalin					
Contact time	Mean virus titer [log_10_ TCID_50_/g]	Titer reduction [log_10_ TCID_50_/g]	Detectable by titration	Detectable in passage 1	Detectable in passage 2
Liver—Ebola virus					
Untreated pre-purification [n = 6]	7.24 [6.01–7.60]	−	−	−	−
Untreated post-purification [n = 6]	6.63 [5.21–7.50]	−	Yes (3/3)	Yes (3/3)	−
10 min [n = 6]	≤ 1.89 [1.69–2.07]	≥ 4.38 [3.40–5.44]	No (0/3)	No (0/3)	No (0/3)
1 h [n = 3]	≤ 1.74 [1.69–1.80]	≥ 4.52 [3.66–5.78]	No (0/3)	No (0/3)	No (0/3)
3 days [n = 3]	≤ 1.76 [1.58–1.93]	≥ 4.50 [3.88–5.73]	No (0/3)	No (0/3)	No (0/3)
7 days (3 + 4) [n = 3]	≤ 1.77 [1.66–1.94]	≥ 4.47 [3.80–5.79]	No (0/3)	No (0/3)	No (0/3)
Lung—Nipah virus					
Untreated pre-purification [n = 6]	6.40 [5.91–6.99]	−	−	−	−
Untreated post-purification [n = 6]	6.42 [5.84–6.96]	−	Yes (3/3)	Yes (3/3)	−
10 min [n = 6]	≤ 1.75 [1.62–2.01]	≥ 4.56 [4.23–5.34]	No (0/3)	No (0/3)	No (0/3)
1 h [n = 3]	≤ 1.85 [1.74–1.97]	≥ 4.50 [4.05–4.99]	No (0/3)	No (0/3)	No (0/3)
3 days [n = 3]	≤ 1.71 [1.59–1.77]	≥ 4.65 [4.08–5.37]	No (0/3)	No (0/3)	No (0/3)
7 days (3 + 4) [n = 3]	≤ 1.75 [1.66–1.93]	≥ 4.60 [3.98–5.29]	No (0/3)	No (0/3)	No (0/3)
Spleen—Lassa virus					
Untreated pre-purification [n = 3]	6.17 [5.83–6.59]	−	−	−	−
Untreated post-purification [n = 3]	6.87 [6.57–7.23]	−	Yes (3/3)	Yes (3/3)	−
10 min [n = 3]	4.23 [3.14–5.14]	1.90 [0.97–3.28]	Yes (3/3)	Yes (3/3)	−
1 h [n = 3]	≤ 1.65 [1.60–1.74]	≥ 4.49 [4.09–4.98]	No (0/3)	No (0/3)	No (0/3)
3 days [n = 3]	≤ 1.67 [1.60–1.74]	≥ 4.47 [4.17–4.85]	No (0/3)	No (0/3)	No (0/3)
7 days (3 + 4) [n = 3]	≤ 1.69 [1.66–1.73]	≥ 4.45 [4.16–4.87]	No (0/3)	No (0/3)	No (0/3)
Lung—Lassa virus					
Untreated pre-purification [n = 3]	7.74 [7.06–7.86]	−	−	−	−
Untreated post-purification [n = 3]	7.39 [7.03–8.05]	−	Yes (3/3)	Yes (3/3)	−
10 min [n = 3]	4.27 [4.07–4.42]	3.10 [2.78–3.73]	Yes (3/3)	Yes (3/3)	−
1 h [n = 3]	≤ 1.77 [1.67–1.86]	≥ 5.60 [5.25–6.19]	No (0/3)	No (0/3)	No (0/3)
3 days [n = 3]	≤ 1.86 [1.69–2.00]	≥ 5.51 [5.02–6.17]	No (0/3)	No (0/3)	No (0/3)
7 days (3 + 4) [n = 3]	≤ 1.79 [1.74–1.85]	≥ 5.57 [5.29–6.25]	No (0/3)	No (0/3)	No (0/3)

Tissues (liver, lung, and spleen; ~ 0.1–0.3 g) from experimentally infected rodents were treated with 10% neutral buffered formalin (NBF: 1 volume tissue to 10 volumes buffer) for 10 min, 1 h, 3 days, or 7 days (with a complete buffer change at 3 days). After indicated contact times, tissues were removed from NBF, washed with PBS, and homogenized. To remove residual NBF, clarified homogenate supernatants were purified through Amicon Ultra-0.5 Centrifugal Filter Units [50 kDa]. Purified samples were titrated and blind passaged in parallel (Fig. 3A). Detection of infectious virus by titration was confirmed by reporter protein expression, cytopathic effect, or via immunostaining fixed monolayers. Titers are reported as ≤ when no infectious virus was detected in at least one sample. Titer reductions were calculated by subtracting the titer of treated samples from the titer of untreated controls relative to the weight of each tissue sample. Detection of infectious virus by blind passage was determined by RT-qPCR, with a greater than tenfold increase in vRNA (equivalent to a decrease in C_t_ value >3.3) indicating the presence of infectious virus. −, indicates where data was not assessed.

For MagMAX and TriPure, tissues were homogenized in either inactivant at ratios of 1:4 or 1:10 (w/v) for 1 or 10 min (Fig. 3B). For EBOV, NiV, and LASV, no infectious virus was detected after either 1 or 10 min contact time when treated with MagMAX at a 1:4 or 1:10 ratio, with > 5 log_10_ reductions observed (Table 4). Similar results were observed when tissues were treated with TriPure at 1:4 or 1:10 ratios. For EBOV, NiV, and LASV, > 5 log_10_ reductions were observed, and no infectious virus was detected in any sample, except for a single replicate of LASV-infected spleen treated with TriPure at a 1:4 ratio for 1 min, in which infectious virus was detected in the second passage, indicating a very low level of residual infectivity (Table 5).

**Table 4:** MagMAX Lysis/Binding Solution Concentrate effectively inactivates Ebola, Nipah, and Lassa viruses in tissues.

MagMAX lysis binding solution concentrate					
Ratio, contact time	Mean virus titer [log_10_ TCID_50_/g]	Titer reduction [log_10_ TCID_50_/g]	Detectable by titration	Detectable in passage 1	Detectable in passage 2
Liver—Ebola virus (n = 3)					
Untreated pre-purification	7.73 [7.62–7.81]	−	−	−	−
Untreated post-purification	7.82 [7.76–7.92]	−	Yes (3/3)	Yes (3/3)	−
Ratio: 1 to 10; 1 min	≤ 1.59 [1.24–2.24]	≥ 6.23 [5.53–6.68]	No (0/3)	No (0/3)	No (0/3)
Ratio: 1 to10; 10 min	≤ 1.23 [1.22–1.25]	≥ 6.58 [6.53– 6.67]	No (0/3)	No (0/3)	No (0/3)
Ratio: 1 to 4; 1 min	≤ 1.20 [0.87–1.86]	≥ 6.61 [5.91–7.05]	No (0/3)	No (0/3)	No (0/3)
Ratio: 1 to 4; 10 min	≤ 0.87 [0.84–0.89]	≥ 6.95 [6.87–7.03]	No (0/3)	No (0/3)	No (0/3)
Lung—Nipah virus (n = 3)					
Untreated pre-purification	7.10 [7.02–7.25]	−	−	−	−
Untreated post-purification	6.95 [6.72–7.27]	−	Yes (3/3)	Yes (3/3)	−
Ratio: 1 to 10; 1 min	≤ 1.45 [1.16– 2.04]	≥ 5.49 [5.24– 5.69]	No (0/3)	No (0/3)	No (0/3)
Ratio: 1 to10; 10 min	≤ 1.43 [1.06–2.06]	≥ 5.52 [5.21–5.66]	No (0/3)	No (0/3)	No (0/3)
Ratio: 1 to 4; 1 min	≤ 1.80 [1.79–1.81]	≥ 5.06 [5.46–4.93]	No (0/3)	No (0/3)	No (0/3)
Ratio: 1 to 4; 10 min	≤ 1.81 [1.81–1.83]	≥ 5.02 [5.46–4.91]	No (0/3)	No (0/3)	No (0/3)
Spleen—Lassa virus (n = 3)					
Untreated pre-purification	7.61 [7.44–7.75]	−	−	−	−
Untreated post-purification	7.41 [7.29–7.49]	−	Yes (3/3)	Yes (3/3)	−
Ratio: 1 to 10; 1 min	≤ 1.80 [1.40–2.50]	≥ 5.61 [4.79–6.05]	No (0/3)	No (0/3)	No (0/3)
Ratio: 1 to10; 10 min	≤ 2.12 [1.40–2.50]	≥ 5.29 [4.83–6.05]	No (0/3)	No (0/3)	No (0/3)
Ratio: 1 to 4; 1 min	≤ 1.79 [1.13–2.13]	≥ 5.62 [5.18–6.32]	No (0/3)	No (0/3)	No (0/3)
Ratio: 1 to 4; 10 min	≤ 1.80 [1.14–2.13]	≥ 5.61 [5.16–6.35]	No (0/3)	No (0/3)	No (0/3)

Tissues (liver, lung, and spleen; ~ 0.1–0.3 g) from experimentally infected rodents were homogenized in MagMAX Lysis/Binding Solution Concentrate at a ratio of 1 volume tissue to 10 volumes reagent or 1 volume tissue to 4 volumes reagent. After homogenization, samples were incubated for 1 min or 10 min before being passed through Pierce detergent removal resin to reduce cytotoxicity. Purified samples were titrated and blind passaged in parallel (Fig. 3B). Detection of infectious virus by titration was confirmed by reporter protein expression, cytopathic effect, or via immunostaining fixed monolayers. Titers are reported as ≤ when no infectious virus was detected in at least one sample. Titer reductions were calculated by subtracting the titer of treated samples from the titer of untreated controls relative to the weight of each tissue sample. Detection of infectious virus by blind passage was determined by RT-qPCR, with a greater than tenfold increase in vRNA (equivalent to a decrease in C_t_ value >3.3) indicating the presence of infectious virus. −, indicates where data was not assessed.

**Table 5:** TriPure Isolation Reagent effectively inactivates Ebola, Nipah, and Lassa viruses in tissues.

TriPure isolation reagent					
Ratio, contact time	Mean virus titer [log_10_ TCID_50_/g]	Titer reduction [log_10_ TCID_50_/g]	Detectable by titration	Detectable by passage 1	Detectable by passage 2
Liver—Ebola virus (n = 3)					
Untreated pre-purification	7.73 [7.62–7.81]	−	−	−	−
Untreated post-purification	7.82 [7.76–7.92]	−	Yes (3/3)	Yes (3/3)	−
Ratio: 1 to 10; 1 min	≤ 1.23 [1.20–1.26]	≥ 6.58 [6.52–6.66]	No (0/3)	No (0/3)	No (0/3)
Ratio: 1 to10; 10 min	≤ 1.59 [1.24–2.25]	≥ 6.23 [5.52– 6.68]	No (0/3)	No (0/3)	No (0/3)
Ratio: 1 to 4; 1 min	≤ 0.87 [0.85–0.90]	≥ 6.95 [6.86–7.07]	No (0/3)	No (0/3)	No (0/3)
Ratio: 1 to 4; 10 min	≤ 1.21[0.86–1.82]	≥ 6.61 [5.95–7.06]	No (0/3)	No (0/3)	No (0/3)
Lung—Nipah virus (n = 3)					
Untreated pre-purification	7.17 [7.03–7.42]	−	−	−	−
Untreated post-purification	7.09 [6.85–7.28]	−	Yes (3/3)	Yes (3/3)	−
Ratio: 1 to 10; 1 min	≤ 2.15 [2.13–2.16]	≥ 4.94 [4.72– 5.12]	No (0/3)	No (0/3)	No (0/3)
Ratio: 1 to10; 10 min	≤ 2.15 [2.13–2.16]	≥ 4.94 [4.69– 5.15]	No (0/3)	No (0/3)	No (0/3)
Ratio: 1 to 4; 1 min	≤ 1.16 [0.81–1.84]	≥ 5.93 [5.01–6.45]	No (0/3)	No (0/3)	No (0/3)
Ratio: 1 to 4; 10 min	≤ 1.81 [1.79–1.83]	≥ 5.28 [5.02–5.47]	No (0/3)	No (0/3)	No (0/3)
Spleen—Lassa virus (n = 3)					
Untreated pre-purification	7.81 [7.18–8.35]	−	−	−	−
Untreated post-purification	7.65 [7.29–8.04]	−	Yes (3/3)	Yes (3/3)	−
Ratio: 1 to 10; 1 min	≤ 1.79 [1.43– 2.50]	≥ 5.86 [5.54– 6.18]	No (0/3)	No (0/3)	No (0/3)
Ratio: 1 to10; 10 min	≤ 1.77 [1.40–2.50]	≥ 5.87 [5.54–6.18]	No (0/3)	No (0/3)	No (0/3)
Ratio: 1 to 4; 1 min	≤ 1.45 [1.11–2.11]	≥ 6.20 [5.49–6.91]	No (0/3)	No (0/3)	Yes (1/3)
Ratio: 1 to 4; 10 min	≤ 1.11 [1.09–1.13]	≥ 6.54 [6.20–6.91]	No (0/3)	No (0/3)	No (0/3)

Tissues (liver, lung, and spleen; ~ 0.1–0.3 g) from experimentally infected rodents were homogenized in TriPure Isolation Reagent, at either a 1 volume tissue to 10 volumes reagent, or 1 volume tissue to 4 volumes reagent. After homogenization, samples were incubated for 1 min or 10 min before passing through Pierce detergent removal resin to reduce cytotoxicity. Purified samples were titrated and blind passaged in parallel (Fig. 3B). Detection of infectious virus by titration was confirmed by reporter protein expression, cytopathic effect, or via immunostaining fixed monolayers. Titers are reported as ≤ when no infectious virus was detected in at least one sample. Titer reductions were calculated by subtracting the titer of treated samples from the titer of untreated controls relative to the weight of each tissue sample. Detection of infectious virus by blind passage was determined by RT-qPCR, with a greater than tenfold increase in vRNA (equivalent to a decrease in C_t_ value >3.3) indicating the presence of infectious virus. −, indicates where data was not assessed.

## Discussion

Here, we demonstrate the effective inactivation (≥ 4 log_10_ TCID_50_/g reduction) of EBOV-, NiV-, and LASV-infected tissues by 10% NBF, MagMAX, and TriPure. These data support the use of these reagents for preserving tissue (10% NBF) or nucleic acids (MagMAX/TriPure) while ensuring biosafety in applications such as laboratory research, ecological field studies, or outbreak response. In addition, we identify purification strategies optimized for each reagent that can be broadly applied to inactivation studies of tissues infected with other viral agents. Unlike other methods to reduce cytotoxicity such as dilution, which lowers assay sensitivity, and dialysis, which must be performed over multiple days, disrupts the determination of precise contact time, and can result in a loss of sensitivity, the purification strategizes used here (purification resins or centrifugal filters) can rapidly and effectively reduce cytotoxicity without compromising detection of residual virus^6^.

Inactivation testing does not ensure sterility, as all assays have inherent detection limits. To provide additional assurance in our assays, we evaluated the sensitivity of the methods used here to detect low-level infectious virus. We found that viral titration and cell culture passaging could detect EBOV, NiV, and LASV at titers as low as 0.4–0.7 log_10_ TCID_50_/mL, demonstrating a high level of sensitivity for identifying even low-titer infectious virus. Another limitation in inactivation testing is the magnitude of titers evaluated. Target tissues and selected specimens were chosen to maximize evaluation of inactivation efficacy. Here, we assessed tissues with maximum titers (log_10_ TCID_50_/g) of 6.5–7.8 (EBOV), 5.9–7.4 (NiV), and 6.7–8.4 (LASV). The highest titers we evaluated were greater than 79% of EBOV, 97% of NiV, and all other LASV tissue samples quantified for infectious virus. The only tissue samples with higher titers than those evaluated here were those with insufficient sample volume for testing. However, tissue containing higher titers than those tested here may require longer inactivation periods than those identified as minimally sufficient in this study.

Here, tissues were fixed for up to 7 days in 10% NBF. A key consideration for NBF fixation is specimen size, as samples larger than those tested here may be at risk for incomplete inactivation due to insufficient fixative penetration. For histological analysis, intact tissues are preserved in aldehyde-based fixatives, most commonly 10% NBF (equivalent to ~ 4% formaldehyde). Complete fixation is imperative for inactivation of infectious material prior to transfer to lower containment and for preservation of tissue morphology, but over-fixation can reduce sample quality, diminish immunogenicity, and introduce artifacts^20,21^. Effective fixation requires that NBF fully penetrate to the center of the tissue. Partial penetration may result in inadequate inactivation of infectious material in the sample. To achieve complete fixation, samples are typically limited to ≤ 1 cm^3^, with many guidelines recommending a maximum thickness of 3–5 mm^22,23^. NBF penetrates tissue at an estimated rate of 1–2.5 mm per hour^24,25^, but this can vary with fixative concentration, temperature, pH, and tissue density^26^. In this study, tissues were at least 1 cm in one dimension, but the inherent anatomy of rodent organs typically resulted in thicknesses of < 1 cm. Consequently, adherence to fixation guidelines is inherently feasible for small animal tissues such as those evaluated here, whereas sampling from larger species requires more deliberate attention to ensure adequate fixation.

Other factors relevant to our evaluation of NBF-mediated inactivation include the use of archived tissues that had undergone a freeze–thaw cycle and the homogenization of samples after fixation. As tissues were selected from frozen archives, it is possible that freeze–thaw cycles facilitated more rapid formalin penetration than would occur in fresh tissues. In addition, to assess inactivation throughout the entire specimen, tissues were homogenized after the designated contact time. To minimize the effects of residual formalin, which could facilitate additional inactivation during homogenization, samples were thoroughly washed in PBS and then homogenized in approximately 10 volumes of media per 1 volume of tissue to further dilute any remaining fixative.

Previous studies assessing 10% NBF-mediated inactivation of NiV, EBOV, and LASV in tissues have reported effective inactivation at 2, 7, and 20 days, respectively^7,12,13,17,19^. EBOV was also shown to be inactivated with 2% glutaraldehyde at 7 days^7^. We demonstrate NBF inactivation in as little as 10 min for EBOV and NiV and 1 h for LASV. Unique to our work was the evaluation of shorter time frames, which are rarely reported. One report evaluated a 1 day treatment of NiV with 10% NBF, demonstrating effective inactivation in 3 of 4 (75%) liver samples dissected to 2 cm × 1 cm × 1.5 cm^19^. The differences in reported efficacy are most likely attributable to fixation of tissues with dimensions much greater than those assessed in this study. Collectively, these data highlight the importance of evaluating sample sizes necessary for the application intended. The tissue dimensions evaluated here were sufficient for applications such as the preservation of nucleic acids for diagnostic or pathogenesis studies and for histological analyses.

Notably, our data demonstrates that rapid and effective inactivation occurred well before the multi-day fixation timeframes prescribed in many commonly used guidelines, which often include fixative changes as an additional precautionary measure to help ensure complete inactivation. Consistent with prior reports showing agent-specific differences in resistance to various inactivation methods, including gamma irradiation^2^, photoactivated methylene blue^27^, aldehydes^28^, and alcohols^29^, we also observed agent-specific resistance to 10% NBF. Breakthrough was detected for LASV, but not EBOV or NiV, at the shortest contact time evaluated (10 min). Given the brevity of this exposure time, some breakthrough is not unexpected and may reflect intrinsic agent-specific differences in resistance to formalin inactivation; however, we cannot exclude the possibility that tissue-specific factors contributed to this observation, as LASV samples originated from a different rodent species. Overall, these findings underscore the need to evaluate inactivation efficacy under the specific experimental conditions being used to ensure complete and reliable pathogen inactivation.

For inactivation of tissue compatible with molecular assays, previous studies have shown that TRIzol (phenol and guanidine isothiocyanate; Invitrogen) treatment for 10 min, or Buffer RLT (guanidine isothiocyanate, Qiagen) treatment for 10 min followed by the addition of ethanol for 20 min, effectively inactivates EBOV-infected tissues^7,12^, while a 20 min TRIzol treatment effectively inactivates NiV-infected tissue^13^. These findings, together with our results, support the use of guanidinium-thiocyanate- and phenol-based reagents for the inactivation of high-consequence viral pathogens. Overall, the data generated in this study provide valuable guidance for the safe handling of potentially infectious tissues and support the use of 10% NBF, MagMAX, and TriPure to enable transfer of samples to lower containment for analysis. These findings can also inform risk assessments and biosafety protocols aimed at protecting personnel handling samples in laboratory settings, during outbreak response, or in ecological surveillance.

## Material and methods

### Biosafety

All work with infectious material was conducted in a biosafety level 4 laboratory at the Centers for Disease Control and Prevention (CDC) following established standard operating procedures. All recombinant virus work was approved by the CDC Institutional Biosafety Committee. Animal tissue was derived from studies approved by the CDC Institutional Animal Care and Use Committee (#3220, 2798, 3199, 3129, 2833). Work was performed in an AAALAC International-approved facility, conducted in accordance with the Guide for the Care and Use of Laboratory Animals, and designed and reported in accordance with ARRIVE guidelines.

### Cells

Cell lines were commercially sourced from the American Type Culture Collection (ATCC). Vero (ATCC, Cat. No. CCL-81) and Vero E6 (Vero C1008; ATCC, Cat. No. CRL-1586) cells were maintained in Dulbecco’s minimal essential medium (DMEM; Gibco) supplemented with 5% fetal bovine serum (FBS; Cytiva Hyclone), 1× non-essential amino acids (Gibco), penicillin (100 U/mL)-streptomycin (100 μg/mL) (Gibco), 1 mM sodium pyruvate (Thermo Fisher Scientific), and 2 mM l-glutamine (Thermo Fisher Scientific).

### Origin of virus-infected tissue

Virus inactivation studies were performed using archived tissue specimens from experimental infection studies in CD-1 mice (mouse-adapted EBOV variant Mayinga), Syrian hamsters (NiV- Malaysia and NiV-Bangladesh), and strain 13/N guinea pigs (LASV Josiah) conducted between 2019–2024. Virus information is detailed in Table S1. Target tissues (EBOV–liver, spleen, gonads, eye, brain; LASV–liver, spleen, lung; NiV–liver, kidney, lung, brain) were harvested at euthanasia or study completion and stored at − 80°C. Approximate infectious tissue load from each sample was determined using tissue culture infectious dose 50% (TCID_50_) and quantified using the Reed-Muench method to identify tissues with high titers that can be used for inactivation testing^30^. Tissues were prioritized for inactivation testing if titers exceeded the minimum required threshold and sufficient archived volume was available.

### Reagents for virus inactivation

Reagents used for viral inactivation in this study were: 10% neutral buffered formalin (NBF; Sigma, Cat. No. HT501320), MagMAX Lysis/Binding Solution Concentrate (MagMAX; Thermo Fisher Scientific, Cat. No. AM8500), and TriPure Isolation Reagent (TriPure; Roche, Cat. No. 11667165001). 10% NBF contains formaldehyde (≥ 1.0– < 5.0%, CAS: 50-00-0) and methanol (≥ 1.0– < 3.0%, CAS: 67-56-1). MagMAX contains guanidinium thiocyanate (55–80%; CAS: 593-84-0) and an ionic detergent (proprietary information, concentration not stated). TriPure contains phenol (≥ 30– < 50%, CAS: 108-95-2) and guanidinium thiocyanate (≥ 20– < 30%, CAS: 593-84-0).

### Evaluation of cytotoxicity removal with purification resins and centrifugal filters

The use of purification resins (Pierce detergent removal resin [PDR, Thermo Fisher Scientific, Cat. No. 87780], Sephacryl S400HR [S400, Sigma, Cat. No. S400HR], Sephadex LH20 [LH20, Sigma, Cat. No. LH20100], and Bio-Beads SM-2 Adsorbents [SM2, Bio-Rad, Cat. No. 1523920]) and centrifugal filter units (Amicon Ultra-0.5 Centrifugal Filter Unit, 50 kDa [Amicon, Sigma, Cat. No. UFC5050]) have proven effective at significantly reducing cytotoxic compounds from inactivant-treated cell culture supernatants^6^. To determine if these methods were also suitable for use with tissue samples, tissues (liver and lung) collected from naïve Syrian hamsters (Envigo; HsdHan:Aura, Cat No. 8903M/8903F) were treated with inactivation product at the highest concentration to be assessed (1 volume tissue to either 10 volumes 10% NBF, 4 volumes MagMAX, or 4 volumes TriPure) and incubated for the longest equivalent contact time (10% NBF, 7 days; MagMAX, 10 min; TriPure, 10 min). Tissue samples treated with 10% NBF were washed three times in PBS before homogenization. Tissue samples treated with MagMAX or TriPure were homogenized in the inactivation product as previously described. Purification resins were prepared according to the manufacturer’s instructions, and 0.1 mL homogenate was applied directly to the resin before centrifugation for 2 min at 1000 × g. For centrifugal filter units, sample homogenate was clarified by centrifugation for 10 min at 14,000 × g, and 0.5 mL clarified sample was applied to the filter before centrifugation for 10 min at 14,000 × g. Filters were washed three times in PBS. Purified material was collected in 0.5 mL VI-media supplemented with 10% FBS. Pre- and post-purification material was titrated twofold onto Vero-E6 cells. Cell viability was assessed after 72 h using Cell-Titer Aqueous One Cell Proliferation Reagent (Promega, Cat. No. G3582). Cytotoxic concentration (CC_20_) was defined as the concentration resulting in 80% cell viability.

### Virus titration

Tissue sections (approximately 0.1–0.3 g) were homogenized in 1 mL of viral isolation media (VI-media; DMEM supplemented with 2× antimycotic/antibiotic [Gibco]) using 4 mL polycarbonate grinding vial containing pre-cleaned 3/8″ 440C stainless steel grinding balls (OPS Diagnostics, Cat. No. PCVS 04-240-03). Homogenization was performed using a 2010 Geno/Grinder (SPEX SamplePrep) set to 1500 strokes/minute for 2 min. Tissue samples were clarified by centrifugation (10,000 × g for 10 min) before titrating tenfold onto Vero E6 cells (EBOV) or Vero cells (NiV and LASV). Virus titrations were performed using 12 technical replicates, using 50 µL per replicate. Virus titers (TCID_50_) were determined using the Reed-Muench method^30^ by fluorescence (expressed reporter protein; NiV and EBOV), cytopathic effect (NiV), or indirect immunofluorescence (IFA; EBOV and LASV). For IFA, fixed cell monolayers were permeabilized using 0.1% Triton X-100 and probed using pooled polyclonal rabbit anti-EBOV serum (1:500; CDC SerHarv #703371), or pooled mouse monoclonal anti-LASV ascitic fluid (1:1000; CDC SerHarv #SPR628). Virus titrations were performed identically to assess assay limit of detection and to assess virus inactivation.

### Detection of infectious virus through blind passage

Cell-culture flasks (25 cm^3^) were seeded with 5 × 10^5^ cells and incubated overnight. For limit of detection analysis, monolayers were inoculated with working stocks (0.4 mL) serially diluted to approximately 6.0–0.1 log_10_ TCID_50_/mL, in duplicate. To assess virus inactivation, monolayers were inoculated with all remaining sample available after titration (approximately 0.4–0.5 mL). Flasks were incubated at 37°C, 5% CO_2_ for 7 days. Supernatant (1 mL) from flasks negative for virus replication was blind-passaged into fresh flasks prepared as described above.

Monolayers were examined for cytotoxic effects. Virus replication was assessed by the detection of vRNA by collection of samples at day 0 (D0) and day 7 (D7) from each passage. Cell culture supernatants (100 µL) were collected in duplicate into 400 µL MagMAX Lysis/Binding Solution concentrate with isopropanol (1:1), and RNA was extracted using the MagMAX Pathogen RNA/DNA Kit (Thermo Fisher Scientific, Cat. No. 4462359) on the KingFisher Apex System (Thermo Fisher Scientific). Samples were treated with DNaseI and eluted in 75 µL elution buffer. RNA was stored at -80°C before analysis by RT-qPCR using the SuperScript III Platinum One-Step qRT-PCR kit (Thermo Fisher Scientific, Cat. No. 11732088). Viral RNA was detected using assays designed against the nucleoprotein of EBOV^31^, NiV^32^, or LASV^33^, with an assay targeting *Ppia*^34^ as an internal control. Virus replication was defined as a decrease in C_t_ values greater than 3.3 from D0, observed across both replicates.

### Neutral buffered formalin tissue inactivation

Archived tissue samples (liver, lung, or spleen), stored at -80°C, were thawed, divided into pieces of ≤ 1 cm^3^ (approximately 0.1–0.3 g), and added to 1:10 (w/v) 10% NBF, or as an untreated control, PBS. For each condition (tissue and contact time), tissues from three to six individual animals were used. After contact times of 10 min, 1 h, 3 days, or 7 days (with complete product replacement on day 3), samples were transferred to clean tubes and washed with > 50 mL of PBS to reduce levels of NBF on the tissue surface before adding to polycarbonate grinding vials containing 2 mL VI-media supplemented with 10% FBS. Tissue was homogenized, and homogenate was clarified by centrifugation at 14,000 × g for 10 min. To remove cytotoxic compounds, 0.5 mL clarified supernatant was applied to Amicon Ultra-0.5 Centrifugal Filter Units [50 kDa] (Fig. 3A) and purified as previously described. Each sample was purified in duplicate (1 mL total). Virus titration and blind passaging were performed, as described, to determine if any infectious virus remained after inactivation. Untreated control samples were titrated pre- and post-purification to evaluate virus loss during purification steps. For 10% NBF inactivated samples and equivalent controls, where two centrifugal filters were used per sample, each individual column was titrated separately, with the remaining material pooled for passaging.

### MagMAX Lysis/binding solution concentrate and TriPure isolation reagent tissue inactivation

Archived, infected tissue samples (liver, lung, or spleen) stored at − 80°C were thawed, trimmed to approximately 0.1–0.3 g, and treated with MagMAX or TriPure at tissue-to-reagent ratios of 1:4 or 1:10 (w/v). Untreated controls were prepared at tissue-to-reagent ratios of 1:10 with PBS. For each condition (tissue, inactivant, ratio, and contact time), tissues from three individual animals were used. All samples were immediately homogenized. After contact times of 1 min or 10 min, 1.0 mL homogenized sample was transferred to pre-rinsed 4 mL PDR resin spin columns (Thermo Fisher Scientific, Cat. No. 87779), centrifuged at 1000 × g for 2 min, and eluate collected (Fig. 3B). Virus titration and blind passaging were performed, as described, to determine if any infectious virus remained after inactivation. Untreated control samples were titrated pre- and post-purification to evaluate virus loss during purification steps.

### Graphing and statistical analysis

All graphs were created in GraphPad Prism (v10). The LOD is defined as the lowest concentration of infectious virus that would be detectable in 95% of replicates. The LOD for the TCID_50_ assay was calculated using a probit curve fit to the percentage of positive wells (the hit rate)^35^. The minimum detectable concentration (MDC) is defined as the lowest concentration of infectious virus that could be detected in the TCID_50_ assay, where at ≥ 1 replicate out of 12 replicates would be positive. The MDC was calculated using the probit curve to find the point corresponding to the 95% confidence interval predicted to yield one positive replicate out of 12 replicates for the binomial distribution.

## Supplementary Information

Below is the link to the electronic supplementary material.

Supplementary Material 1

## Data Availability

The datasets used and/or analysed during the current study are available from the corresponding author on reasonable request.
