# Supplementary material for: Inactivation of Ebola, Nipah, and Lassa viruses in tissue using neutral buffered formalin, MagMAX lysis/binding solution, or TriPure isolation reagent
Source: Sci Rep. 2025 Dec 31;16:3712. doi: 10.1038/s41598-025-33750-9 (PMC12852802; doi:10.1038/s41598-025-33750-9)
Supplement: Supplementary file 1 — Supplementary Material 1 [file 41598_2025_33750_MOESM1_ESM.pdf]

Supplementary Materials

Inactivation of Ebola, Nipah, and Lassa viruses in tissue using neutral buffered formalin, MagMAX Lysis/Binding Solution, or TriPure isolation reagent

Katherine A. Davies[1,2], Stephen R. Welch[1], Brian H. Harcourt[1], Christina F. Spiropoulou[1], Jessica R. Spengler[1*]

[1]Viral Special Pathogens Branch, Division of High-Consequence Pathogens and Pathology, Centers for Disease Control and Prevention, Atlanta, Georgia, USA
[2]Zoonotic and Emerging Disease Research Unit, National Bio and Agro-Defense Facility, Agricultural Research Service, United States Department of Agriculture, Manhattan, Kansas, USA

*Corresponding author: Jessica R. Spengler; wsk7@cdc.gov

Table S1. Virus strain and modification details for infectious tissue samples used for virus titration and/or inactivation studies.

Sample Details							Samples used for Virus Titration [n]										Samples used for Inactivation Studies [n]		
**Virus Strain**	**Modification**	**GenBank**	**VirHarv**	**Model**	**Model Strain**	**PMID**	**Liver**	**Spleen**	**Testis**	**SV**	**Ovary**	**Uterus**	**Kidney**	**Lung**	**Eye**	**Brain**	**Liver**	**Spleen**	**Lung**
EBOV (Mayinga)	MA	AF499101	813741	Mice	Hsd:ICR CD-1	9728532	8	8	4	3	4	4	–	–	8	8	–	–	–
EBOV (Mayinga)	MA, Rec.	OQ784117	815066	Mice	Hsd:ICR CD-1	37145895	8	8	4	4	4	4	–	–	8	8	1	–	–
EBOV (Mayinga)	MA, Rec., ZsG	OQ784118	815067	Mice	Hsd:ICR CD-1	37145895	8	8	4	4	4	4	–	–	5	7	8	–	–
EBOV (Mayinga)	MA, Rec., NanoLuc	OQ784119	815068	Mice	Hsd:ICR CD-1	37145895	4	4	2	1	2	2	–	–	1	1	–	–	–
EBOV (Mayinga)	MA, Rec., ZsG-NanoLuc	OQ784121	815069	Mice	Hsd:ICR CD-1	37145895	5	5	1	1	4	4	–	–	2	4	–	–	–
EBOV (Mayinga)	MA, Rec., NanoLuc-ZsG	OQ784120	815070	Mice	Hsd:ICR CD-1	37145895	7	7	3	3	4	4	–	–	3	6	3	–	–
NiV (Malaysia)	None	AF212302	813744	Syrian hamster	HsdHan: Aura	11504554	7	–	–	–	–	–	2	9	–	4	–	–	4
NiV (Malaysia)	Rec.	NA	813745	Syrian hamster	HsdHan: Aura	39326503	3	–	–	–	–	–	2	1	–	4	–	–	–
NiV (Malaysia)	Rec., ZsG	NA	813746	Syrian hamster	HsdHan: Aura	39326503	4	–	–	–	–	–	5	5	–	2	–	–	4
NiV (Bangladesh)	Rec., ZsG	NA	815062	Syrian hamster	HsdHan: Aura	39326503	–	–	–	–	–	–	–	7	–	2	–	–	–
NiV (Bangladesh)	Rec., mScarlet-teLuc	PV753716	815111	Syrian hamster	HsdHan: Aura	39326503	–	–	–	–	–	–	–	9	–	–	–	–	3
LASV (Josiah)	Rec.	HQ688675/ HQ688673	813752	Guinea pig	Strain 13/N	21307206	13	13	–	–	–	–	–	5	–	–	–	9	3

Table S1. Virus strain and modification details for infectious tissue samples used for virus titration and/or inactivation studies. –, indicates no samples were tested. EBOV, Ebola virus; LASV, Lassa virus; MA, mouse-adapted; n, number; NA, not applicable; NanoLuc, NanoLuciferase; NiV, Nipah virus; PMID, PubMed Identifier referring to report describing the input virus strain; Rec, recombinant; SV, seminal vesicle; teLuc, teal NanoLuciferase; VirHarv, VSPB reference number; ZsG, ZsGreen1.
